# Characterization of chitosan/alginate/lovastatin nanoparticles and investigation of their toxic effects *in vitro* and *in vivo*

**DOI:** 10.1038/s41598-020-57666-8

**Published:** 2020-01-22

**Authors:** Hoang Thai, Chinh Thuy Nguyen, Loc Thi Thach, Mai Thi Tran, Huynh Duc Mai, Trang Thi Thu Nguyen, Giang Duc Le, Mao Van Can, Lam Dai Tran, Giang Long Bach, Kavitha Ramadass, C. I. Sathish, Quan Van Le

**Affiliations:** 1 Institute for Tropical Technology, Vietnam Academy of Science and Technology, 18, Hoang Quoc Viet, Cau Giay, Hanoi 100000 Vietnam; 2 Vinh, University, 182 Le Duan, Vinh, Nghe An 460000 Vietnam; 3 Department of Functional Exploration, Military Hospital 103, 261 Phung Hung, Phuc La, Ha Dong, Hanoi 100000 Vietnam; 4 Department of Pathophysiology, Vietnam Military Medical University, 160 Phung Hung, Phuc La, Ha Dong, Hanoi 100000 Vietnam; 5 Graduate University of Science and Technology, Vietnam Academy of Science and Technology, 18 Hoang Quoc Viet, Cau Giay, Hanoi 100000 Vietnam; 6 NTT Institute of High Technology, Nguyen Tat Thanh University, 300A Nguyen Tat Thanh, District 4, Ho Chi Minh City, 700000 Vietnam; 7 Global Innovative Center for Advanced Nanomaterials, Faculty of Engineering and Built Environment, The University of Newcastle, Callaghan, 2308 NSW Australia

**Keywords:** Biochemistry, Biochemistry, Carbohydrates, Carbohydrates

## Abstract

In this study, chitosan and alginate were selected to prepare alginate/chitosan nanoparticles to load the drug lovastatin by the ionic gelation method. The synthesized nanoparticles loaded with drug were characterized by Fourier transform infrared spectroscopy (FT-IR), scanning electron microscopy (SEM), laser scattering and differential scanning calorimetry (DSC) methods. The FTIR spectrum of the alginate/chitosan/lovastatin nanoparticles showed that chitosan and alginate interacted with lovastatin through hydrogen bonding and dipolar-dipolar interactions between the C-O, C=O, and OH groups in lovastatin, the C-O, NH, and OH groups in chitosan and the C-O, C=O, and OH groups in alginate. The laser scattering results and SEM images indicated that the alginate/chitosan/lovastatin nanoparticles have a spherical shape with a particle size in the range of 50–80 nm. The DSC diagrams displayed that the melting temperature of the alginate/chitosan/lovastatin nanoparticles was higher than that of chitosan and lower than that of alginate. This result means that the alginate and chitosan interact together, so that the nanoparticles have a larger crystal degree when compared with alginate and chitosan individually. Investigations of the *in vitro* lovastatin release from the alginate/chitosan/lovastatin nanoparticles under different conditions, including different alginate/chitosan ratios, different solution pH values and different lovastatin contents, were carried out by ultraviolet-visible spectroscopy. The rate of drug release from the nanoparticles is proportional to the increase in the solution pH and inversely proportional to the content of the loaded lovastatin. The drug release process is divided into two stages: a rapid stage over the first 10 hr, then the release becomes gradual and stable. The Korsmeyer-Peppas model is most suitable for the lovastatin release process from the alginate/chitosan/lovastatin nanoparticles in the first stage, and then the drug release complies with other models depending on solution pH in the slow release stage. In addition, the toxicity of alginate/chitosan/lovastatin (abbreviated ACL) nanoparticles was sufficiently low in mice in the acute toxicity test. The LD_50_ of the drug was higher than 5000 mg/kg, while in the subchronic toxicity test with treatments of 100 mg/kg and 300 mg/kg ACL nanoparticles, there were no abnormal signs, mortality, or toxicity in general to the function or structure of the crucial organs. The results show that the ACL nanoparticles are safe in mice and that these composite nanoparticles might be useful as a new drug carrier.

## Introduction

Currently, for the treatment of obesity (due to cholesterol in the blood exceeding the recommended limit) and cardiovascular disease, lovastatin is a commonly used drug that is effective and harmless. However, its applications are limited because of the low bioavailability in the major cycle and short half-life (approximately 3 hr), etc. Therefore, using polymer composites to load lovastatin can help to control the drug release ability to overcome the limitations of lovastatin in the treatment of the abovementioned diseases. The physical interactions between the polymers and lovastatin (hydrogen bonding, dipole-dipole interactions, etc.), facilitate the absorption and distribution of lovastatin to more easily control its release.

There are reports on the synthesis of polymer composites based on chitosan and polycaprolactone that have been used as carriers for lovastatin. These investigations mainly reported lovastatin release and established a suitable kinetic equation for drug release in solutions with different pH values^1–5^. It has been reported that lovastatin/polymer nanoparticles could be effective in sustaining drug release for a prolonged period^3^.

Recently, interest in investigating polymer nanoparticles made from alginate and chitosan as drug carriers has been increasing due to their good adhesion, biodegradability, and biocompatibility^6–11^. Alginate particles are quite sensitive to pH, swelling in alkaline solutions and shrinking in acidic solutions.

When a drug is coated with alginate, the drug will be protected from the effects of the gastric acid environment and be deliver directly into the intestine. Alginate particles could be the best suitable drug carriers for oral drug manufacturing. However, alginate is hydrophilic, and it is difficult to arrange alginate particles in an oil-water phase during the preparation process, leading to unstable emulsions. Therefore, alginate particles are mixed with chitosan to improve the hydrophilic properties of alginate but still retain the biological compatibility and pH sensitivity of alginate^12^. It is widely known that chitosan has high biodegradability and biocompatibility, and the amino groups of the chitosan molecules can interact with cations to form a cationic polymer and easily create complexes or nanoparticles in water to carry drug molecules. A porous structure and large specific surface area help chitosan load drugs with high capacity^13^. The combination of the carboxyl groups of alginate and the protonated amino groups of chitosan can form a polymer-electrolyte complex (PEC). The formation of the PEC limits the disadvantages of chitosan, such as its poor mechanical durability and unstable structure after implantation/transplantation, hydrophobicity, and reduced moisture stability while retaining the biological activity of the material, leading to the formation of new materials with better properties and the ability to control drug and protein release more easily^14,15^. Alginate-chitosan microspheres and nanospheres have been synthesized by an emulsion technique, suspension or ionic gelation method and have been applied to load model drugs, such as rifampicin^7^, nifedipine^8^, 5-fluorouracil^9^, curcumin diethyl disuccinate^11^, insulin^16^, ampicillin^17^, curcumin^18^, verapamil^19^, trimetazidine hydrochloride (TH)^20^, doxorubicin and paclitaxel^21^, and paclitaxel^22^. The obtained results indicated that polymers are required to control the drugs in solutions of different pH values.

In our previous papers, alginate/chitosan polymer blend films (with an alginate/chitosan ratio of 8/2) carrying lovastatin (10, 20 and 30 wt.% calculated by comparison with the total alginate and chitosan weight) or ginsenoside Rb1 were extracted from Vietnamese *Panax pseudoginseng* (5, 10, 15 and 20 wt.% calculated by comparison with the total alginate and chitosan weight) and prepared by the solution method at room temperature^23,24^. The results demonstrated that the alginate/chitosan films contributed to the controlled release of lovastatin and ginsenoside Rb1 from the alginate/chitosan film-loaded drugs at various solution pH values.

Published papers have only focused on the preparation of micro- and nanosized polymer composites based on alginate and chitosan carrying drugs as well as the drug release *in vitro* and drug release kinetics in different pH solutions. However, there have been no reports thus far on the fabrication of alginate (AG)/chitosan (CS) particles on the nanoscale carrying lovastatin by ionic gelation methods and investigation into their special properties. Therefore, this study concentrated on the preparation of alginate/chitosan/lovastatin nanoparticles using an ionic gelation method and investigation of the interactions between alginate and lovastatin, and chitosan and lovastatin, and the effects of the alginate/chitosan (AG/CS) ratio on the distribution and properties of the alginate/chitosan/lovastatin nanoparticles. In addition, the *in vitro* lovastatin release and drug release kinetics from the alginate/chitosan/lovastatin (ACL) nanoparticles in different pH solutions are considered and discussed. An acute toxicity evaluation of the alginate/chitosan/lovastatin nanoparticles was carried out *in vivo* in adult healthy Swiss mice. The suitable AG/CS ratio of 6.5/3 (wt.%/wt.%) was best for the preparation of the ACL nanoparticles by the ionic gelation method. The ACL nanoparticles formed successfully in the range of 50–100 nm. Our *in vivo* results show that the ACL nanoparticles are safe, nontoxic and might be applied to lower serum cholesterol in animal models as well as humans.

## Experimental

### Materials and methods

Sodium alginate (AG) powder, viscosity 300–500 mPa.s; chitosan (CS) powder with deacetylation degree >75–85%, polymer density index 1.61 × 10^5^ Da; and lovastatin (LS) powder, purity ≥98.0% were products of Sigma Aldrich Co. (St. Louis, MO 63103 USA). KCl, NaOH, KH_2_PO_4_, HCl (37 wt %), ethanol, and acetic acid (purity 99.5%) were analytical reagents that were used as received without further purification.

### Preparation of AG/CS/LS nanoparticles

The AG/CS/LS nanoparticles were prepared by ionic gelation according to the following steps: First, AG was dissolved in distilled water until a solution was formed before the addition of CaCl_2_ to increase the viscosity of the solution (solution 1). In addition, CS was dissolved in 1% acetic acid solution (solution 2), while LS was dissolved in ethanol (solution 3). Next, solution 1 was added dropwise to solution 2 and stirred in an ultrasonic bath to form a uniform solution. Thereafter, solution 3 was poured into the mixture of solution 1 and solution 2 and then ultrasonicated five times for 5 mins. Finally, the mixed solution was centrifuged at 4 °C before lyophilization in a FreeZone 2.5 machine (Labconco, USA). The ratios of AG, CS, LS, and the coding of prepared samples are presented in Table 1.

**Table 1:** The ratios of AG, CS and LS and the signature and diameter (d) the of prepared samples.

AG (wt.%)	CS (wt.%)	LS (wt.%)	Sample code	d (nm) (±S.D.)
60.6	30.4	9.0	AC6/3-L10	900.8 ± 101.1
62.2	28.8	9.0	AC6.5/3-L10	86.2 ± 3.7
63.6	27.3	9.0	AC7/3-L10	220.2 ± 17.5
57.0	26.3	16.7	AC6.5/3-L20	91.3 ± 10.0
52.6	24.3	23.1	AC6.5/3-L30	490.0 ± 18.8

### Characterization

The mean diameter and size distribution of the nanoparticles were measured by a Zetasizer Ver 6.20 particle size analyzer (Malvern Instruments Ltd.). Fourier transform infrared spectroscopy (FTIR) spectra of the ACL nanoparticles were recorded on a Nicolet/Nexus 670 spectrometer (USA) at room temperature with 32 scans at 8 cm^−1^ resolution and wavenumbers ranging from 400 to 4000 cm^−1^. Field emission scanning electron microscopy (FESEM) images of the LS and ACL nanoparticles were conducted using an S-4800 field emission scanning electron microscope (Hitachi). The thermal properties of the LS, AG, CS and ACL nanoparticles were determined on a DSC-60 thermogravimetric analyzer (Shimadzu) under an argon atmosphere from room temperature to 600 °C at a heating rate of 10 °C/min.

### *In vitro* drug release study

The *in vitro* LS release tests were carried out on ACL nanoparticles as follows: 15 mg of each sample was immersed in 100 ml of phosphate-buffered saline (PBS) (pH 6.5 and 7.4), HCl/KCl buffer solution (pH 2) or acetic acid/sodium acetate buffer solution pH (4.5) at 37 °C and placed in an incubated shaker at 120 rpm. Over 30 hr at predetermined time intervals, 5 ml aliquots were withdrawn, and the concentration of LS released was monitored by a UV spectrophotometer (CINTRA 40, GBC, USA). The dissolution medium was replaced with fresh solution to maintain the total volume. The LS release percent can be determined by the following equation:

$${\rm{Drug}}\,{\rm{release}}\,[ \% ]={{\rm{C}}}_{({\rm{t}})}/{{\rm{C}}}_{(0)}\times 100$$

where C_(0)_ and C_(t)_ represent the amount of LS loaded and the amount of drug released at time t, respectively. All studies were performed in triplicate.

### Drug release kinetic study

According to the review literature, there are different types of drug release kinetics and mechanisms based on the polymer matrix. Drug release from a polymer matrix usually implies water penetration in the matrix, hydration, swelling, diffusion of the dissolved drug, and/or erosion of the gelatinous layer. It is worth mentioning that the release mechanism of a drug depends on the drug dose, investigation of the solution pH, and the nature of the drug and polymer used. Recently, the most probable kinetics have been used for drug release, as depicted below^1,2,20^:

Zero-order kinetics

1 $${{\rm{W}}}_{{\rm{t}}}={{\rm{W}}}_{0}+{{\rm{k}}}_{1}{\rm{t}}$$

First-order kinetics

2 $$\log \,{{\rm{C}}}_{{\rm{t}}}=\,\log \,{{\rm{C}}}_{0}-{{\rm{k}}}_{2}{\rm{t}}/2.303$$

Hixson-Crowell’s cube-root equation (erosion model)

3 $${(100-{\rm{W}})}^{1/3}={100}^{1/3}-{{\rm{k}}}_{3}{\rm{t}}$$

Higuchi’s square root of time equation (diffusion model)

4 $${{\rm{W}}}_{{\rm{t}}}={{\rm{k}}}_{4}{\rm{t}}$$

Power law equation (diffusion/relaxation model)

5 $${{\rm{M}}}_{{\rm{t}}}/{{\rm{M}}}_{\infty }={{\rm{k}}}_{5}{{\rm{t}}}^{{\rm{n}}}$$

where k is the drug release constant; C_t_ and C_o_ are the concentrations of drug at the initial time and testing time; W_t_ and W_o_ are the weights of the drug at the initial time and testing time; M_t_/M_∞_ is the fractional drug release into the dissolution medium; and n is the diffusional constant that characterizes the drug release transport mechanism. When n ≤ 0.5, the drug diffusion from the polymer matrix corresponds to a Fickian diffusion or a quasi-Fickian diffusion mechanism. When 0.5 < n < 1, an anomalous, non-Fickian drug diffusion occurs. When n = 1, a non-Fickian, case of II (relaxational) transport or zero-order release kinetics could be observed, and when n >1, super case II transport was observed.

To find the kinetic models for the LS release process from the ACL nanoparticles, the drug release data obtained from section 2.4 were calculated according to the 5 above kinetic equations.

### Acute and subchronic toxicity of the alginate/chitosan/lovastatin nanoparticles

Adult healthy Swiss mice were housed in individual cages with a 12 hr light/dark cycle and fed food and water ad libitum. Animals were fasted overnight prior to drug injection. The mice were treated in strict compliance with The Animal Center Guidelines for the Care and Use of Laboratory Animals at the Vietnam Military Medical University, consistent with the EU Directive 2010/63/EU. This study was approved by the Committee for Animal Experiments and Ethics (Institutional Animal Care and Use Committee, IACUC) at the Vietnam Military Medical University.

#### Toxicity evaluation

The acute toxicity in the present study was conducted as per the top-down procedure of the Organization for Economic Cooperation and Development (OECD) guideline with minor modifications^25^.

(i) Limit test
  Initially, the ACL nanoparticles were injected into two animals at a single dose of 2000 mg/kg by oral gavage. Animals were observed once during the first 3–4 hr after the injection and then periodically during the first 72 hr. As the two animals were not dead, the drug was administered to two additional animals at a single dose of 5000 mg/kg.
(ii) Main test

Forty adult Swiss mice were randomly divided into 4 groups with 10 animals per group. The animals in each group were injected with a single dose of 500 mg/kg, 1000 mg/kg, 2000 mg/kg or 5000 mg/kg. All the animals were observed daily for any abnormal behavior and mortality for 72 hr. Thereafter, histological changes in the animal livers and kidneys were examined.

#### Subchronic toxicity

Repeated-dose oral subchronic toxicity of the ACL nanoparticles was carried out according to OECD guideline 407^26^. Animals were randomly divided into 3 groups with 10 animals per group. Group 01 (control): rats were treated with saline; groups 02 and 03: animals were injected with ACL nanoparticles at doses of 100 mg/kg and 300 mg/kg (containing 10 mg/kg and 30 mg/kg lovastatin), respectively. Animals were administered saline or lovastatin at a dosage of 0.1 ml per 10 g body weight daily by gavage. After the injection, pharmacotoxic signs and mortality of the animals were observed at 10 min, 30 min, 60 min, and 4 hr during the first day and daily thereafter for 28 days. The body weights of all animals were recorded weekly before and after dosing. On the 28^th^ day, blood samples were collected from all animals via abdominal aorta puncture for biochemical and hematological analyses. Hematological parameters, including the number of white blood cells (WBCs), number of blood cells (RBCs), hemoglobin (g/dL), hematocrit (%), MCV (fl), MCH (pg), MCHC (%), and platelet (10^3^/µL), were determined using a Sysmex XN-1000 analyzer (Japan). Biochemical parameters including alanine aminotransferase (ALT), aspartate aminotransferase (AST), creatinine, and urea were analyzed using a fully automated biochemical analyzer (AU 640- Olympus, Japan). On the 29^th^ day, all animals were injected with ketamine at a dose of 30 mg/kg, and their kidneys and livers were excised, weighed, and examined macroscopically. Thereafter, the livers and kidneys were preserved in 10% buffered formalin for histopathological study.

#### Data analysis

The body weights, biochemical and hematological indexes were analyzed by repeated two-way ANOVAs following Tukey’s test for multiple comparisons. Significant differences were defined at p < 0.01. All data are expressed as the mean ± SEM.

## Results and Discussion

### The size distribution of the ACL nanoparticles

**Figure 1:**
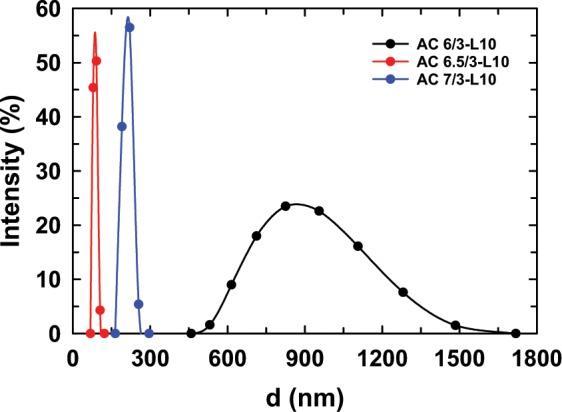
Size distribution diagram of the ACL nanoparticles prepared with different AG/CS ratios.

To investigate the influence of the AG/CS ratio on the size distribution of ACL nanoparticles, the ACL samples were prepared with different AG/CS ratios by ionic gelation, as listed in Table 1. Figure 1 shows the size distribution diagrams of the AC6/3-L10, AC6.5/3-L10, and AC7/3-L10 samples. The particle sizes of all the samples ranged from 68 nm to 1718 nm. Among the prepared samples, the sample composed of an AG/CS ratio of 6.5/3 had the smallest particles with an average diameter of approximately 86.2 ± 3.7 nm (see Table 1). In our opinion, the different size distribution of the tested samples might be caused by the diverse interactions between the solvent and drug, the drug and drug, the solvent and polymer, the polymer and polymer, the solvent and solvent, the drug and polymer, and so on. In general, smaller particles are better for dissolution of drugs into solution. Therefore, a suitable AG/CS ratio for the preparation of the ACL nanoparticles is 6.5/3.

**Figure 2:**
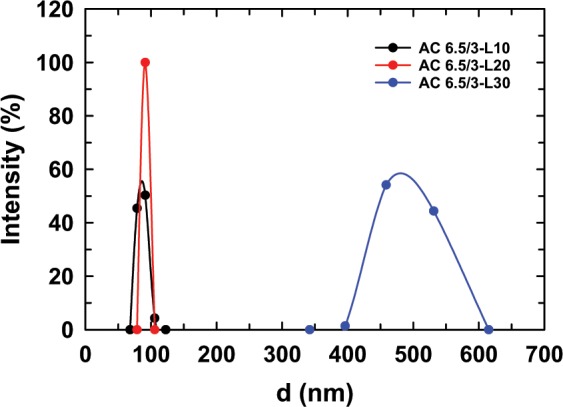
Size distribution diagram of the ACL nanoparticles prepared with different LS contents.

The ACL nanoparticles were prepared with the LS content in the range of 10 to 30 wt.% in comparison with the total weight of AG and CS to investigate the effect of LS content on the size of ACL the nanoparticles (Fig. 2). It was clearly observed that the size of the ACL nanoparticles with LS contents of 10 wt.% and 20 wt.% were much smaller than that of the ACL nanoparticles containing 30 wt.% LS (Table 1). This means that using a low content of LS can form ACL nanoparticles with a smaller particle size. This can be explained as follows: at a small concentration of LS, the interactions between the drug and polymer are stronger than the interactions between the drug and drug. Therefore, polymers can absorb a large amount of drug and the drug can be distributed regularly in the ACL nanoparticles, resulting in ACL nanoparticles having a tighter structure and smaller size. In contrast, when using a high concentration of LS, the drug-drug interactions predominate and are stronger than the drug-polymer interactions. Thus, LS can agglomerate in the structure of the ACL nanoparticles and cannot be absorbed completely into the polymers, causing the larger ACL nanoparticle size and LS remaining in solution. As mentioned in other reports, the average size of CS/AG particles for loading drugs is from nanosized to microsized depending on the ratio of CS/AG and the nature of the drug^11,14,16–18^. For example, chitosan/alginate nanoparticles carrying curcumin diethyl disuccinate (CDD) have an average particle size from 440 to 695 nm^11^, and alginate-chitosan-pluronic F127 nanoparticles loading curcumin have a particle size of approximately 100 ± 20 nm^18^.

### Morphology of the ACL nanoparticles

**Figure 3:**
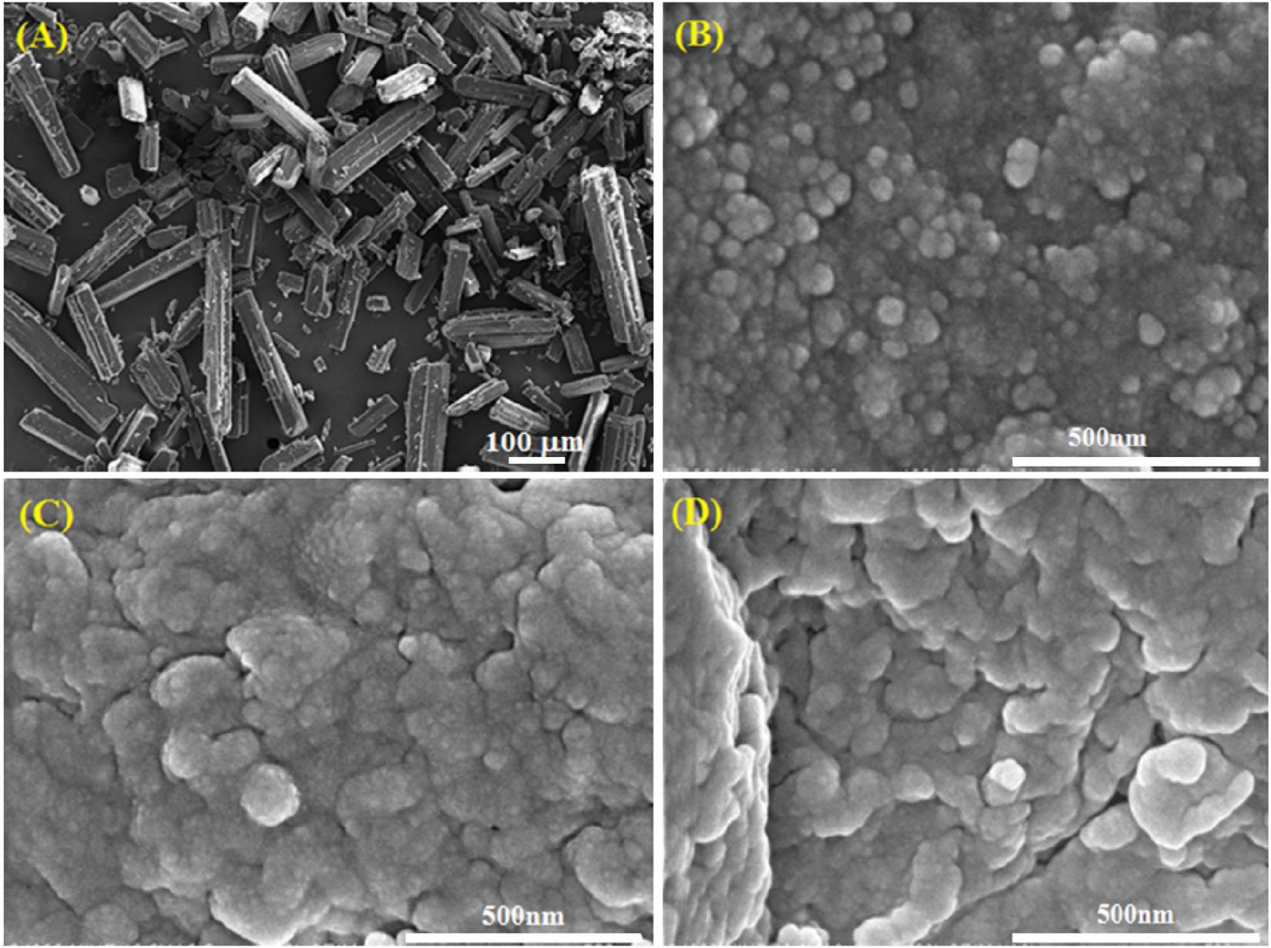
SEM images of the (**A**) lovastatin; (**B**) AC6.5/3-L10; (**C**) AC6.5/3-L20; and (**D**) AC6.5/3-L30 samples.

The FE-SEM images of the LS and ACL nanoparticles with different contents of LS are shown in Fig. 3. It is clear that LS is bar-shaped with an irregular size from 150–500 µm (Fig. 3A). The AC6.5/3-L20 and AC6.5/3-L30 nanoparticles have a tendency to agglomerate together from 100–200 nm sized particles to form larger particles on the micro scale (Fig. 3C,D). Interestingly, the AC6.5/3-L10 nanoparticles are spherical, separate and uniformly sized, at only 50–80 nm (Fig. 3B). This result is in agreement with the size distribution results mentioned above. Predictably, at 10 wt.% of LS, the LS can interact stronger with polymers and distribute better in the CS and AG. This contributes to the improvement of LS dissolution in buffer solutions.

### FTIR spectra of the ACL nanoparticles

**Figure 4:**
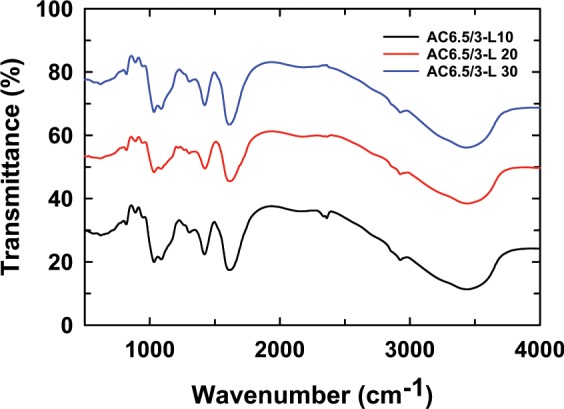
FTIR spectra of the ACL nanoparticles prepared with different LS contents.

Figure 4 presents the FTIR spectra of the ACL nanoparticles prepared with different LS contents. It is clearly visible that there is a slight shift of some characteristic peaks in the spectrum of ACL nanoparticles when compared with the FTIR spectra of LS, CS, and AG, as reported in our previous paper^21^. The stretching vibrations of the C=O groups of AC6.5/3-L10, AC6.5/3-L20, and AC6.5/3-L30 nanoparticles are assigned to 1608.27 cm^−1^, 1616.23 cm^−1^ and 1623.41 cm^−1^, respectively. This can be described by the difference in the wavenumber of the C=O group in the FTIR spectrum of the ACL nanoparticles in comparison with those of CS, AG and LS. In particular, the peaks corresponding to the NH_2_, C-O, and OH groups in the FTIR spectrum of the ACL nanoparticles are also shifted significantly (3–105 cm^−1^) in comparison with similar peaks in the FTIR spectra of AG, CS and LS. These changes can be caused by interactions between the C=O, C–O, and OH groups of LS with the C–O, NH, and OH groups of CS and the C=O, C–O, and OH groups in AG through dipole-dipole interactions and hydrogen bonds (as assumed in Table 2)^7,27,28^. A wide peak at approximately 2200 cm^−1^ in the FTIR spectrum of the ACL nanoparticles corresponds to the presence of the -NH_3_C group in the polyion complex formed by the electrostatic interaction of dissociated carboxylate groups of sodium alginate with protonated amino groups from chitosan^29,30^. In addition, the change in LS content has less of an effect on the structure of the ACL nanoparticles because the wavenumber of some of the characterized groups shifts slightly, between 1–7 cm^−1^.

**Table 2:** Predicted hydrogen bonding interactions of AG, CS and LS.

Materials	Type of hydrogen bonding
LS	LS–C=O**…**H–O–LS
LS and AG	LS–O–H**…**O=C–AG
	LS–C=O**…**H–O–AG
LS and CS	LS–C=O**…**H–O–CS
	LS–C=O**…**H–N–CS
	LS–O–H**…**O(H)–CS
	LS–O–H**…**N(H)–CS
CS and CS	CS–N–H**…**O(H)–CS
	CS–O–H**…**N(H)–CS
AG and AG	AG–O–H**…**O=C–AG
CS and AG	AG–C=O**…**H–O–CS
	AG–C=O**…**H–N–CS
	AG–O–H**…**O(H)–CS
	AG–O–H**…**N(H)–CS

### Thermal behavior of the ACL nanoparticles

**Figure 5:**
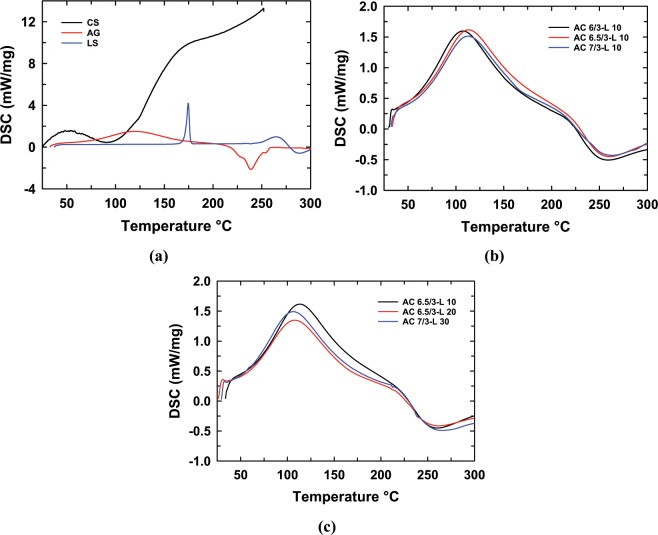
DSC diagrams of the CS, AG, LS (**a**), ACL nanoparticles with different AG/CS ratios (**b**) and ACL nanoparticles with different LS contents (**c**).

Figure 5 and Table 3 display the DSC diagrams and DSC data of LS, CS, AG and the ACL nanoparticles with different LS concentrations. The melting temperatures of LS, CS and AG were 174.6 °C, 106.8 °C and 119.7 °C, respectively, while the melting temperature of the ACL nanoparticles was 107.2 °C–113.4 °C, depending on the LS content and the AG/CS ratio (Table 3 and Fig. 5). Noticeably, the melting temperature of the ACL nanoparticles was higher than that of CS and lower than that of AG. This result confirms that CS and AG are partly compatible through some physical interactions, as mentioned in Table 2. The interaction and compatibility between AG and CS contribute to the formation of a stable structure of ACL nanoparticles, and these nanoparticles could better control drug release. From the data listed in Table 3 and Fig. 5b, the nanoparticles prepared at an AG/CS ratio of 6.5/3 have the highest melting temperature, corresponding to AG and CS in AC6.5/3-L10 nanoparticles. These nanoparticles could be more compatible than the AC6/3-L10 and AC7/3-L10 nanoparticles. In addition, the LS content also affects the interaction and compatibility of AG and CS due to the interactions between the drug and polymer or the drug and drug in the nanoparticle system. Table 3 and Fig. 5c show the differences in the melting temperatures and melting enthalpies of the ACL nanoparticles with different LS contents. These results can be explained by the weak interaction between LS and CS and AG at the large LS content, leading to an ACL nanoparticle structure that is less tight and melts more easily. As a result, the AC6.5/3-L20 and AC6.5/3-L30 nanoparticles have melting temperatures and melting enthalpies lower than that of the AC6.5/3-L10 nanoparticles.

**Table 3:** Melting temperature (T_m_) and melting enthalpy (ΔH_m_) of AG, CS, LS and the ACL nanoparticles.

Sample	T_m_ (°C)	ΔH_m_ (J/g)
AG	119.7	358.6
LS	174.6	90.3
CS	106.8	130.6
AC6/3-L10	107.6	590.4
AC7/3-L10	112.6	501.3
AC6.5/3-L10	113.4	545.2
AC6.5/3-L20	107.5	467.4
AC6.5/3-L30	107.2	541.3

### Drug release study

#### Effect of the AG/CS ratio on drug release content from the ACL nanoparticles

**Figure 6:**
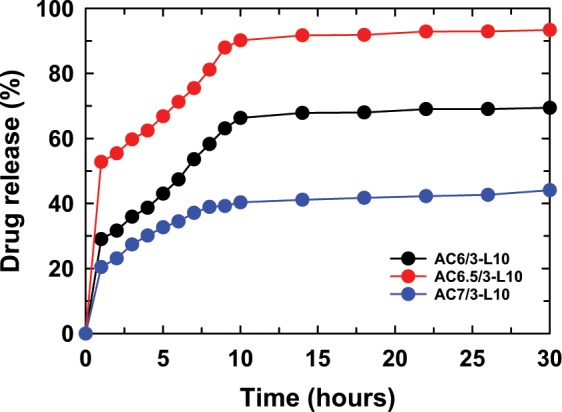
LS released from the ACL nanoparticles prepared with different AG/CS ratios in solution at pH 7.4.

The results of the effects of the AG/CS ratio on the LS release content from the ACL nanoparticles tested in a pH 7.4 solution are shown in Fig. 6. Interestingly, LS is quickly released from the nanoparticles in the initial 10 hr and then the release is slower or/and controlled. The AG/CS ratio remarkably influences the drug release content from ACL nanoparticles. For instance, more than 50 wt.% of the drug is released from the AC6.5/3-L10 nanoparticles after testing for 2 hr. This result is similar to AG/CS nanoparticles loaded with other drugs, such as 5-fluorouracil (5-FU), tegafur, and verapamil^9,19^. After testing for 30 hr, the LS released from the ACL nanoparticles at pH 7.4 reached 93.38%, 69.45% and 44.18%, corresponding to the AC6.5/3-L10, AC6/3-L10, and AC7/3-L10 samples, respectively. The reason LS releases from the AC6.5/3-L10 sample more quickly than from the AC6/3-L10, and AC7/3-L10 nanoparticles might be due to the smaller particle size of the AC6.5/3-L10 sample. Due to the smaller particle size, the nanoparticles can dissolve and diffuse into the buffer solution more easily, releasing LS from the nanoparticles faster.

#### Investigation of the effect of solution pH on drug release from the ACL nanoparticles

**Figure 7:**
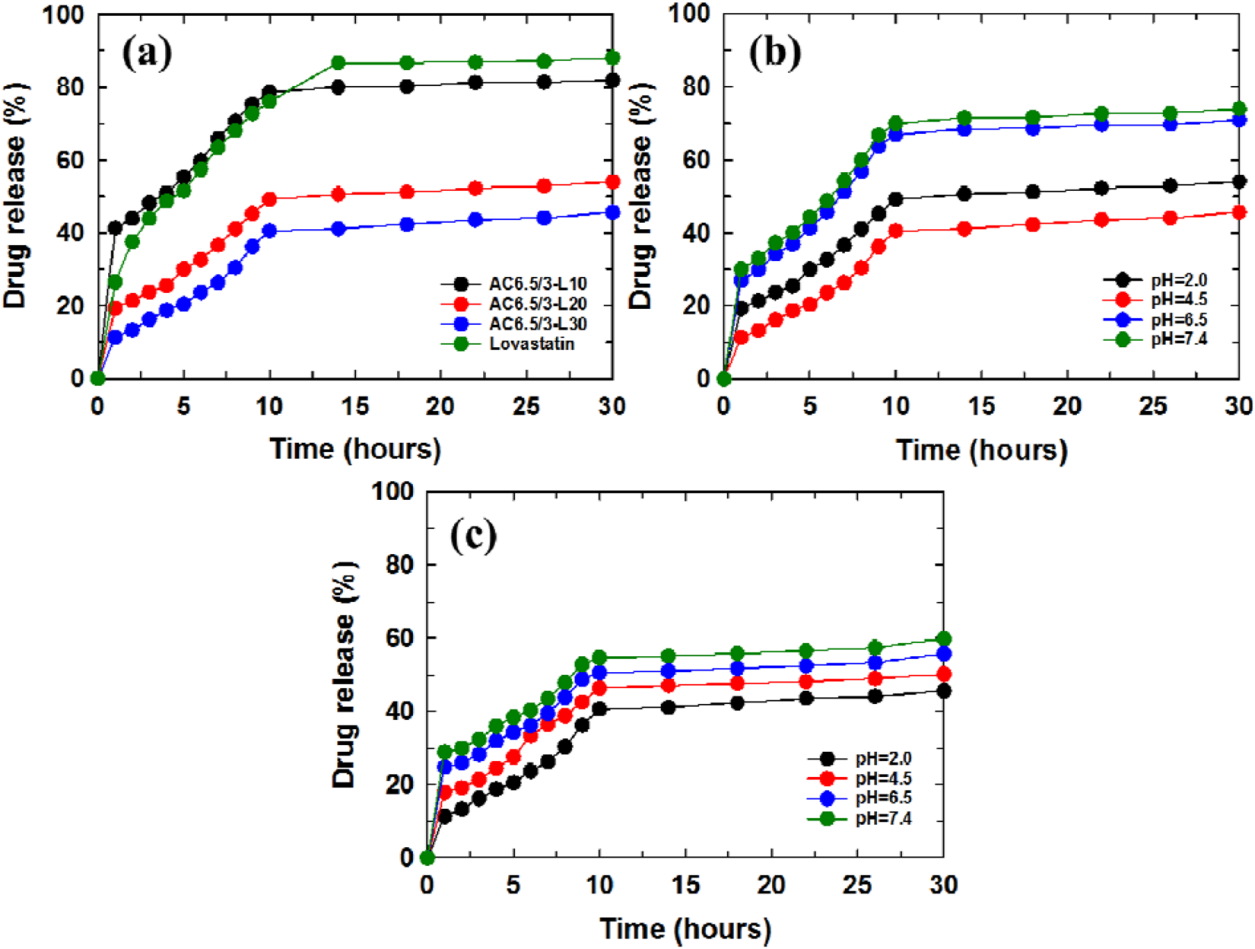
LS released from (**a**) AC6.5/3-L10, (**b**) AC6.5/3-L20 and (**c**) AC6.5/3-L30 nanoparticles in different pH solutions.

Figure 7 presents the LS content released from the AC6.5/3-L10 nanoparticles in solutions of different pH values (pH 2.0, 4.5, 6.5, and 7.4). The LS content released from AC6.5/3-L10 nanoparticles in different pH solutions, such as 2.0, 4.5, 6.5, and 7.4, displayed that the solution pH strongly affected the drug release content. After 30 hr of testing, the LS content released from the AC6.5/3-L10 nanoparticles at pH 7.4, 6.5, 4.5 and 2.0 corresponded to 93.38%, 90.44%, 84.15% and 83.04%, respectively. It can be concluded that the LS release content from AC6.5/3-L10 nanoparticles in different pH solutions is on the order of pH 7.4 > pH 6.5 > pH 4.5 > pH 2.0. The results obtained from the AC6.5/3-L20 and AC6.5/3-L30 nanoparticles were similar to the release values of LS from the AC6.5/3-L10 nanoparticles. The LS released from these samples also followed the order pH 7.4 > pH 6.5 > pH 4.5 > pH 2 (Fig. 7b,c). The faster drug release from the ACL nanoparticles in the neutral environment compared with that in an acidic environment could be caused by the repulsion between H^+^ ions (in acidic pH) and cations on the surface of the CS, inhibiting the hydrolysis of the polymer^4,20,31^. These results indicate that the ACL nanoparticles are better suited for the basic environment of the large intestine, colon and rectal mucosa than an acidic environment.

#### Effect of the initial LS content on drug release content from the ACL nanoparticles

**Figure 8:**
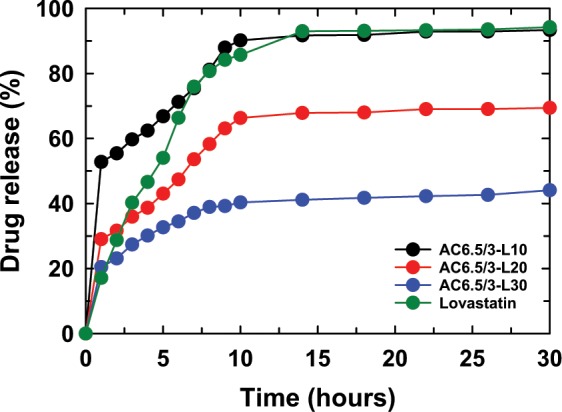
LS released from the ACL nanoparticles in solution at pH 7.4.

**Figure 9:**
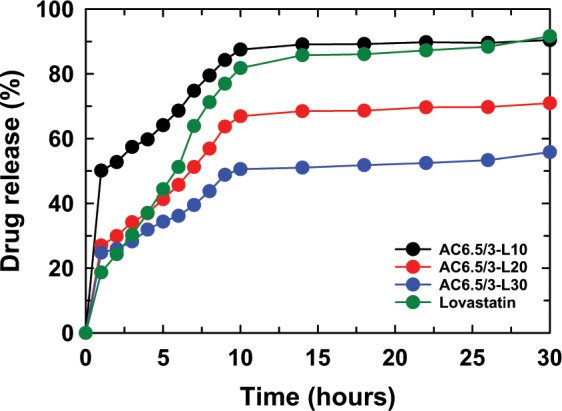
LS released from the ACL nanoparticles at pH 6.5.

**Figure 10:**
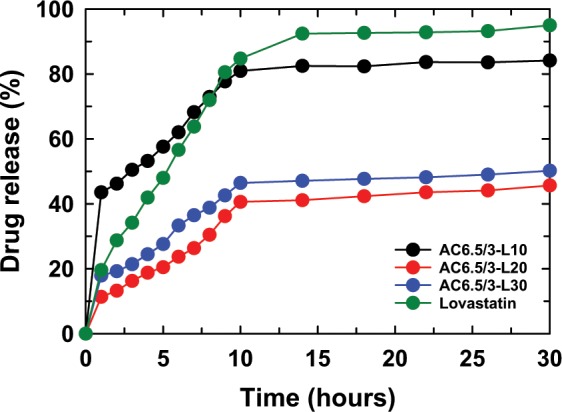
LS released from the ACL nanoparticles at pH 4.5.

**Figure 11:**
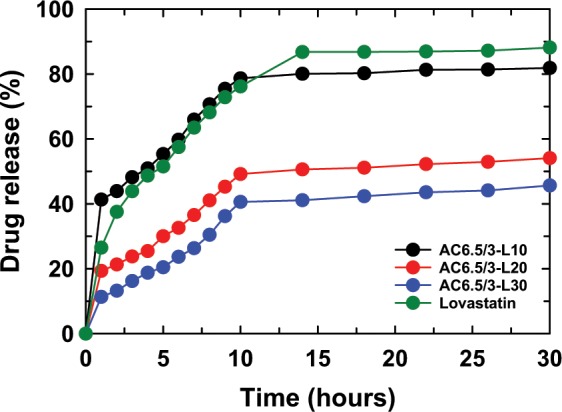
LS released from the ACL nanoparticles at pH 2.

Figures 8–11 show the LS content released from the control sample and ACL nanoparticles prepared using different LS concentrations in different buffer solutions. It was found that the ACL nanoparticles containing a high LS content during the initial phase of the test period released less LS when compared with the ACL nanoparticles having a low LS concentration, where the release of LS from the ACL particles was higher. This drug release pattern occurred at all tested pH values, including 7.5, 6.5, 4.5 and 2. The particle size of the ACL nanoparticles increased as the LS concentration increased; therefore, smaller nanoparticles have a faster drug release ability, as mentioned above. The drug release in the high LS-loaded ACL nanoparticles was slower due to the existence of more free void space between larger-sized nanoparticles and LS; therefore, drug molecule transport and diffusion of the drugs into solution became more difficult. Comparing the LS control sample and the AC6.5/3-L10 nanoparticles in solutions of different pH, it is clear that at the initial stage, the LS released from the AC6.5/3-L10 nanoparticles is remarkably faster than the release from the LS sample without nanoparticle loading. It is interesting to note that, at pH 7.4, the drug released from the AC6.5/3-L10 nanoparticles after 1 hr of testing was 52.8%, which was much higher than the LS control sample, where the drug release was only 17.1% (Fig. 8). The same drug release pattern was observed at all other tested pH values of 6.5, 4.5 and 2 over the same one hour testing duration. This confirms that LS loaded in the AG/CS nanoparticles can dissolve and diffuse more easily into the buffer solution than the drug that was not loaded into the nanoparticle. The AG/CS blend can be used as a suitable carrier for LS to improve its dissolution.

### Drug release kinetic study

**Figure 12:**
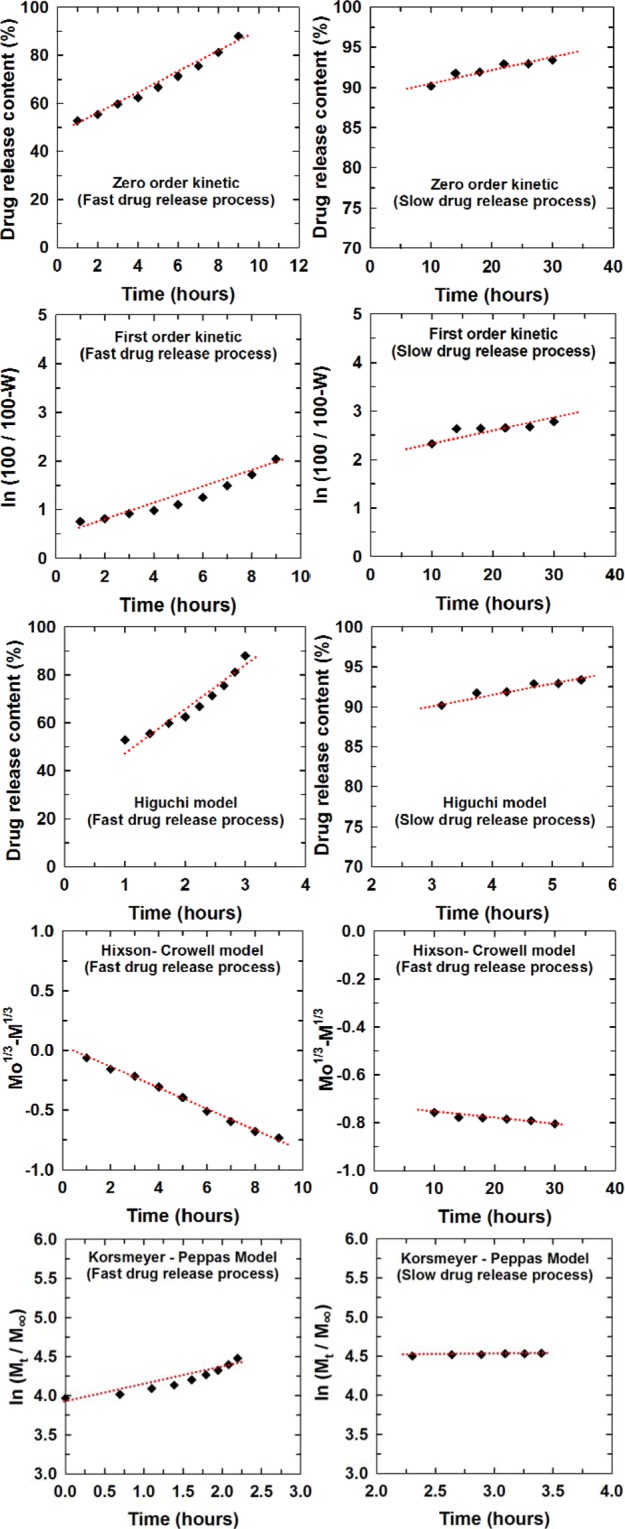
Kinetic equations fit to different models (Eqs. 1–5 in section 2.5), reflecting LS release from the AC6.5/3-L10 nanoparticles in pH 7.4 solution.

The kinetic equation and regression coefficients (R^2^) of the LS release process from the AC6.5/3-L10 nanoparticles in solution at pH 7.4 are displayed in Fig. 12. The regression coefficient value from the Hixson-Crowell model, is the most appropriate for studying the LS release kinetics from AC6.5/3-L10 nanoparticles in solution pH 7.4 at the first stage (R^2^ = 0.996), while the Korsmeyer-Peppas model (diffusion/relaxation model) is the most suitable for studying the LS release kinetics from the AC6.5/3-L10 nanoparticles at pH 7.4 at the controlled stage (R^2^ = 0.947). This result means that in the fast release stage for the first 9 hr, the mechanism for the release of LS from the AC6.5/3-L10 nanoparticles into the pH 7.4 solution resulted from the system having a change in the surface area and particle size. This suggested that the dissolution of the drug occurred together with the dissolution of alginate in the AC6.5/3-L10 nanoparticles, leading to a decrease in the diameter of the nanoparticles. For the controlled stage or slow stage from 10 to 30 hr, the suitability of the Korsmeyer-Peppas model (R^2^ = 0.947) for LS release from the AC6.5/3-L10 nanoparticles into pH 7.4 solution proves that the drug mechanism was a combination of many processes, including the swelling and dissolution of the polymers, the diffusion and dissolution of the drug, and so on. These results comply with the LS release from the other polymers in other literature reports^1,2,20^.

The regression coefficients of the LS release process for the AC6.5/3-L10 nanoparticles in solutions of different pH (6.5, 4.5 and 2.0) according to the different kinetic models were also determined and are reported in Table 4. From the data in Table 4, it is clear that the fast drug release and slow drug release processes comply with different kinetic models in different pH solutions. For the initial stage, LS released from the AC6.5/3-L10 nanoparticles follows the Korsmeyer-Peppas model with the highest regression coefficients corresponding to pH values of 6.5, 4.5 and 2.0, which were 0.997, 0.989, and 0.998, respectively. Therefore, it can be concluded that the LS release from AC6.5/3-L10 nanoparticles at the initial stage is complex. In the subsequent stage, LS release from the AC6.5/3-L10 nanoparticles is simpler, except for the solution at pH 2.0. For instance, in the solutions at pH 6.5 and 4.5, the LS release process from the AC6.5/3-L10 nanoparticles complied with the zero order kinetic model and first order kinetic model. In the case of the solution at pH 2.0, the dissolution of CS in the acidic environment caused a remarkable effect on the LS release process, leading to a complex drug release mechanism.

**Table 4:** Regression coefficients obtained from the kinetic equations reflecting LS release from AC6.5/3-L10 nanoparticles in solutions of different pH values (ZO: zero order kinetic model; FO: first order kinetic model; HG: Higuchi model; HCW: Hixson-Crowell model; KMP: Korsmeyer- Peppas model).

Solution	Drug release process (fast)				
	ZO	FO	HG	HCW	KMP
pH 7.4	0.985	0.932	0.935	0.996	0.888
pH 6.5	0.957	0.987	0.938	0.965	0.997
pH 4.5	0.967	0.989	0.914	0.899	0.989
pH 2.0	0.983	0.966	0.927	0.919	0.998
**Solution**	**Drug release process (slow)**				
	**ZO**	**FO**	**HG**	**HCW**	**KMP**
pH 7.4	0.889	0.707	0.923	0.925	0.947
pH 6.5	0.990	0.921	0.924	0.932	0.983
pH 4.5	0.954	0.971	0.967	0.931	0.956
pH 2.0	0.965	0.968	0.970	0.905	0.979

### Acute toxicity

According to the Organization for Economic Cooperation and Development – OECD guideline No. 423^25^, a compound/material that possesses an LD_50_ greater than 5000 mg/kg b.w. could be assigned as a Class 5 substance and be considered nontoxic or less toxic. From the results of the limit test and the main acute toxicity studies, all animals from the experimental groups did not appear to have any obvious symptoms of toxicity. After drug administration for 72 hr, two animals with a single dose of 2000 mg/kg and four animals with a single dose of 5000 mg/kg were not dead. It is clear that the median lethal dose (LD_50_) of the ACL nanoparticles is higher than 5000 mg/kg^25^.

### Subchronic toxicity

During a 28 day period of testing, three rat groups were used for the drug loading study (with and without the use of 100 mg/kg or 300 mg/kg ACL nanoparticles), and there were no serious abnormal signs or mortality found. Table 5 shows that the body weights of rats in three rat groups increased naturally throughout the treatment period (0–28 days).

**Table 5:** Mean body weights of the three rat groups (without and with the use of ACL nanoparticles) after the 28 day treatment period.

Week	Control (g, ± SEM)	ACL nanoparticle groups (g, ± SEM)	
		Dose of 100 mg/kg	Dose of 300 mg/kg
0	185.16 ± 13.68	176.00 ± 14.20	198.33 ± 13.78
1	187.16 ± 13.78	176.66 ± 14.30	199.66 ± 13.77
2	195.00 ± 15.66	188.83 ± 16.26	206.50 ± 15.89
3	198.33 ± 16.53	196.66 ± 17.12	213.83 ± 16.65
4	205.16 ± 17.73	205.00 ± 18.32	223.00 ± 17.74

Table 6 displays the hematological parameters of the three rat groups without and with ACL nanoparticle treatment after a 28 day treatment period. It is clear that there were no significant differences in the hematological parameters between these rat groups.

**Table 6:** Hematological parameters of the three rat groups without and with ACL nanoparticles after the 28 day treatment period.

Parameter	Control (mean ± SEM)	ACL nanoparticle groups (mean ± SEM)	
		Dose of 100 mg/kg	Dose of 300 mg/kg
WBC (10^3^/µl)	8.01 ± 0.863	8.24 ± 0.916	8.19 ± 0.866
RBC (10^6^/µl)	6.66 ± 0.189	6.83 ± 0.125	6.19 ± 0.313
Hemoglobin (g/dL)	11.88 ± 0.295	12.09 ± 0.279	10.88 ± 0.465
Hematocrit (%)	36.48 ± 0.979	36.68 ± 1.037	33.08 ± 1.352
MCV (fl)	54.02 ± 0.885	53.27 ± 0.886	53.67 ± 0.692
MCH (pg)	17.88 ± 0.190	17.55 ± 0.238	17.67 ± 0.186
MCHC (%)	32.66 ± 0.276	33.05 ± 0.304	32.94 ± 0.249
Platelets (10^3^/µl)	481.80 ± 46.490	368.90 ± 65.180	468.90 ± 68.120

Table 7 demonstrates the biochemical parameters of the three rat groups with and without the use of ACL nanoparticles after the 28 day treatment period. There were no significant differences in the biochemical parameters between these rat groups.

**Table 7:** Biochemical parameters of the three rat groups with and without the use of ACL nanoparticles after the 28 day treatment period.

Parameter	Control (mean ± SEM)	ACL nanoparticle groups (mean ± SEM)	
		Dose of 100 mg/kg	Dose of 300 mg/kg
AST (U/L)	153.31 ± 11.92	198.03 ± 46.84	158.10 ± 19.82
ALT (U/L)	38.22 ± 3.849	53.95 ± 12.63	45.94 ± 4.708
Creatinine (mg/dL)	65.42 ± 2.769	71.16 ± 1.909	60.31 ± 4.629
Urea (%)	4.64 ± 0.392	4.16 ± 0.231	4.98 ± 0.397

After the 28 day treatment period, the organs of all rats in the three experimental groups were excised, weighed, and examined macroscopically. Table 8 shows the weights of some of the important organs from the rats. The results using one-way ANOVA software indicated that there were no significant differences in the liver, kidney, and spleen weights between the three tested rat groups (p > 0.05).

**Table 8:** Mean weights of the rat organs in the three rat groups without and with ACL nanoparticles after the 28 day treatment period.

Organ	Control group (g, ± SEM)	ACL nanoparticle groups (g, ± SEM)	
		Dose of 100 mg/kg	Dose of 300 mg/kg
Liver	6.43 ± 0.28	6.32 ± 0.44	6.53 ± 0.58
Kidney	1.29 ± 0.099	1.248 ± 0.058	1.402 ± 0.116
Spleen	0.332 ± 0.023	0.342 ± 0.025	0.354 ± 0.028

**Figure 13:**
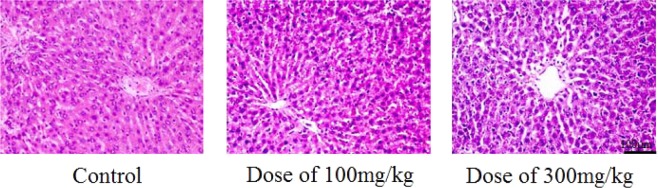
Histological changes in the livers of rats (without and with ACL nanoparticles) after the 28 day treatment period.

**Figure 14:**
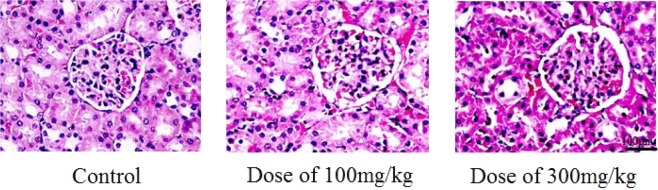
Histological changes in the kidneys of the three rat groups (without and with the use of ACL nanoparticles) after the 28 day treatment period.

Histological changes in the livers of three tested rat groups with and without ACL nanoparticles after the 28 day treatment period were normal, while fatty degeneration in hepatocytes was observed in both ACL nanoparticle-treated rat groups (Fig. 13). However, the kidney sections of these rat groups after the 28 day treatment period indicated hemorrhage in the glomerulus and interstitial tissue (Fig. 14).

Histopathology of the liver: bright-field image of an H&E stained section showing the normal hepatic architecture and the components of basic liver lobules with the portal area. Liver cells were arranged in a funicular pattern along with central veins and healthy Kupffer cells. The portal area contained no inflammatory cells. The scale bar is 100 μm.

Histopathology of the kidney: Images of H&E stained sections showing the glomerular and renal vein with edema exudate and a clear Bowman cavity with a normal kidney basement membrane. The vascularity of the cells was normal with relatively healthy tubular epithelial cells. The scale bar is 100 μm.

From the obtained results of the acute and subchronic toxicities for the three rat groups with and without ACL nanoparticles after the 28 day treatment period, the median lethal dose (LD_50_) of the ACL nanoparticles is greater than 5000 mg/kg. According to the classification reported by Diener *et al*. (1995), substances with LD_50_ values of 5000 mg/kg or above are considered practically nontoxic^32^. Thus, there were no symptoms of serious toxicity and mobility, suggesting that the drug is safe in both the acute and subchronic tests. Furthermore, the histopathological investigations of the livers and kidneys showed that administration of the alginate/chitosan/lovastatin nanoparticles induced no serious changes to the histological images of the livers or kidneys.

In summary, AC nanoparticles carrying 10 wt.% lovastatin were tested to evaluate the acute and subchronic toxicities in three groups of rats. The biochemical and hematological parameters and organ function and structural integrity of the rat groups treated with ACL nanoparticles indicated that there were no significant differences after 28 days- of treatment. The obtained results showed that the nanoparticles might not be harmful. Previous studies have indicated that the LD_50_ of lovastatin was higher than 1000 mg/kg^33^. These results are consistent with our previous results from acute toxicity studies. Lovastatin has been used at a dose as low as 10 mg/kg to lower serum cholesterol in rats^34^. Our ACL nanoparticles might be safe, nontoxic or generally less toxic and also not alter the function and structure of some crucial organs in the experimental animals according to OECD guideline 407^26^.

## Conclusions

It has been concluded from this study that a suitable AG/CS ratio for the preparation of ACL nanoparticles by the ionic gelation method is 6.5/3 (wt.%/wt.%). The ACL nanoparticles were synthesized successfully in a size range of between 50–100 nm. FTIR spectra showed that LS, CS and AG interact with each other and change the structure and shape of the ACL nanoparticles. The results of the DSC analysis confirm that CS and AG are partly compatible. The LS release from the AC6.5/3-L10 nanoparticles was higher than that from all other investigated samples at different AG/CS ratios, solution pH values and initial LS contents. The drug release process was divided into two stages: a rapid release in the first 10 hr, then it a gradual and stable release. The Korsmeyer-Peppas model with a complex mechanism is most suitable for the LS release process from ACL nanoparticles in the initial stage, and then, in the slow release stage, drug release complies with the Higuchi model, zero order kinetic model, first order kinetic model or Korsmeyer-Peppas model depending on the solution pH. From the obtained results, it can be confirmed that 10 wt.% LS is appropriate for further application of ACL nanoparticles in treatment. Our *in vivo* results show that ACL nanoparticles are safe, nontoxic and might be applied to lower serum cholesterol in animal models as well as humans.
